# Rate of CRL4^CRBN^ substrate Ikaros and Aiolos degradation underlies differential activity of lenalidomide and pomalidomide in multiple myeloma cells by regulation of c-Myc and IRF4

**DOI:** 10.1038/bcj.2015.66

**Published:** 2015-10-02

**Authors:** C C Bjorklund, L Lu, J Kang, P R Hagner, C G Havens, M Amatangelo, M Wang, Y Ren, S Couto, M Breider, Y Ning, A K Gandhi, T O Daniel, R Chopra, A Klippel, A G Thakurta

**Affiliations:** 1 Translational Development, Celgene Corporation, Summit, NJ, USA; 2 Celgene Corporation, San Diego, CA, USA

## Abstract

Recent discoveries suggest that the critical events leading to the anti-proliferative activity of the IMiD immunomodulatory agents lenalidomide and pomalidomide in multiple myeloma (MM) cells are initiated by Cereblon-dependent ubiquitination and proteasomal degradation of substrate proteins Ikaros (IKZF1) and Aiolos (IKZF3). By performing kinetic analyses, we found that the downregulation or proteasomal degradation of Ikaros and Aiolos led to specific and sequential downregulation of c-Myc followed by IRF4 and subsequent growth inhibition and apoptosis. Notably, to ensure growth inhibition and cell death, sustained downregulation of Ikaros and Aiolos, c-Myc or IRF4 expression was required. In addition, we found that the half-maximal rate, rather than the final extent of Ikaros and Aiolos degradation, correlated to the relative efficacy of growth inhibition by lenalidomide or pomalidomide. Finally, we observed that all four transcription factors were elevated in primary MM samples compared with normal plasma cells. Taken together, our results suggest a functional link between Ikaros and Aiolos, and the pathological dysregulation of c-Myc and IRF4, and provide a new mechanistic understanding of the relative efficacy of lenalidomide and pomalidomide based on the kinetics of substrate degradation and downregulation of their downstream targets.

## Introduction

The seminal observation that thalidomide binds Cereblon (CRBN), a substrate receptor of the cullin ring E3–ubiquitin ligase complex, CRL4^CRBN^, represents a significant breakthrough in our understanding of the pleiotropic activities of IMiD immunomodulatory drugs, including lenalidomide and pomalidomide.^1^ It has been previously postulated that binding to CRBN modulates the E3–ligase complex activity and its preference for substrate selection.^1, 2^ The first validated substrates of the CRL4^CRBN^ complex were shown to be the hematopoietic zinc-finger transcription factors Ikaros (IKZF1) and Aiolos (IKZF3). In the presence of thalidomide, lenalidomide or pomalidomide (Pom) in either multiple myeloma (MM) cells^3, 4^ or T cells,^5^ both Ikaros and Aiolos are ubiquitinated and targeted for degradation by the ubiquitin–proteasome system. Both ubiquitination and subsequent degradation of these proteins are specifically dependent on the presence of CRBN, as either RNA interference silencing or knockout of CRBN abrogates these effects. In addition, Ikaros and Aiolos are essential for the proliferation of MM cell lines *in vitro*, pointing out their potential functional role in malignant plasma cell growth.^3, 4^

Pathological dysregulation of c-Myc and IRF4 are a common feature of MM cells compared with normal plasma cells.^6, 7, 8, 9^ The proposed c-Myc-IRF4-positive autoregulatory loop is only transiently active during the expansion phase after normal B-cell activation.^10, 11, 12, 13, 14^ Subsequently, c-Myc becomes downregulated to allow for differentiation to the plasma cell stage. Consistent with this model, Ikaros and Aiolos have been shown to act as negative regulators of c-Myc expression promoting maturation of normal mouse B-cell development, where Aiolos is necessary for long-lived high-affinity plasma cells.^11, 15, 16^ Previous studies demonstrated that both c-Myc and IRF4 are downregulated by lenalidomide or pomalidomide.^9, 17, 18^ In addition, the observed downregulation of c-Myc and IRF4 in conjunction with the promoted degradation of Ikaros and Aiolos in MM cell lines indicates that IMiD immunomodulatory compounds could directly interfere with the disease-promoting activities of c-Myc and IRF4 via Aiolos and Ikaros.^3, 4, 19, 20^ Although the underlying mechanism is unknown, it is possible that in MM cells Ikaros and Aiolos act as positive regulators of IRF4 and also c-Myc, and that the targeted degradation of these activators in the presence of IMiD drugs leads to the transcriptional downregulation of one or both of these proteins.

In this study we explored how Ikaros and Aiolos may regulate the expression of c-Myc and IRF4 by performing kinetic analysis of protein expression using inducible short hairpin RNA (shRNA) knockdown systems for Ikaros and Aiolos. Our results indicate that the sustained proteasomal degradation or downregulation of Ikaros or Aiolos lead to specific and sequential downregulation of c-Myc protein followed by IRF4 before growth inhibition and apoptosis. Furthermore, we found that the half-maximal rate (*T*_1/2_) rather than the maximal amount (Max_red_) of Ikaros and Aiolos degradation correlate with relative efficacy of growth inhibition by lenalidomide or pomalidomide.

## Materials and methods

### Cell lines

All MM cell lines (ATCC, Manassas, VA, USA) were routinely tested for mycoplasma and maintained as previously described.^21^

### Antibodies

Several antibodies were used for immunoblotting in these experiments including *Ikaros (Millipore, Billerica, MA, USA; Abcam, Cambridge, MA, USA; Santa Cruz Biotechnology, Dallas, TX, USA), Aiolos (Santa Cruz Biotechnology and Celgene developed), IRF4 (Santa Cruz Biotechnology), c-Myc (Abcam) and β-actin (LI-COR Biotechnology, Lincoln, NE, USA). It is noteworthy that several antibodies for both Ikaros and Aiolos exhibited varying degrees of background bands that varied from source and lot-to-lot. In particular, variability was greater for nearly all immunoblots of Ikaros, where there are mostly two bands (only one band for some cell lines) at the predicted molecular weight (likely isoforms) and where sometimes both bands are degraded with drug treatment and others only show degradation of one band.

### Proliferation and viability assays

Cell growth curves were determined by monitoring the viability of cells with Trypan blue exclusion on a Vi-Cell-XR (Becton Dickinson, Franklin Lakes, NJ, USA). Proliferation assays were performed in triplicate at least three times (*n*=3) using (^3^H)-thymidine incorporation as previously described.^18^ All data were plotted and analyzed using GraphPad Prism 5 (GraphPad Software, La Jolla, CA, USA) software, represented as the mean with an error determined as ±s.d.

### Immunoblotting

Immunoblot analysis was performed as previously described at least two times each (*n*⩾2), where the best representative is shown.^18, 21^ Densitometric quantitation of protein bands was analyzed using Image Studio software (LI-COR Biotechnology) and normalized to β-actin loading controls.

### Flow cytometry

Annexin-V Alexa Fluor 488-conjugated antibody (Thermo Scientific, Waltham, MA, USA) and To-Pro-3 (Thermo Scientific) were used according to the manufacturer's protocol and processed as previously described for at least three independent experiments (*n*=3).^22^

### shRNA knockdown

Tetracycline (also doxycycline (DOX))-inducible shRNA constructs targeting either Aiolos, Ikaros, c-Myc or IRF4 (Cellecta, Mountain View, CA, USA) or a Luciferase negative control were generated as lentivirus as previously described.^22^ shRNA lentivirus was then used to transduce vectors into either MM1.S or U266 cells followed by selection with puromycin (Sigma-Aldrich, St Louis, MO, USA). At least two shRNA constructs for each target were used throughout the entirety of these studies, yet for simplicity the data from the first listed shRNA sequence (Supplementary Table S1) for each target are represented in all figures. All shRNA experiments were performed at least three times (*n*=3).

### Kinetics of drug-induced degradation of Ikaros and Aiolos

Following immunoblot analysis and densitometric quantitation and normalization of protein bands to loading and vehicle controls for each time point, relative protein was then transformed into fraction affected (*F*_a_) as the % of relative protein reduced at each time point (*t* in hours) and subsequently fit to a rectangular hyperbolic function.


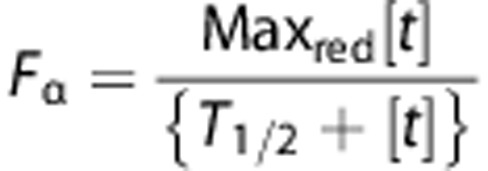


These models were then used to calculate the approximate time for relative reduction in 50% of protein (*T*_1/2_) and the maximal amount of protein reduced (Max_red_).

### Immunohistochemistry

Normal or primary MM bone marrow samples dual stained with either CD138/Aiolos, CD138/Ikaros, CD138/c-Myc or CD138/IRF4 were applied using the Leica Bond automated slide stainer (manuscript in preparation).^23^ Briefly, anti-CD138 mouse monoclonal antibody (Dako, Carpinteria, CA, USA) was used at a 1/1200 dilution and was detected using Bond Polymer Refine Red Detection kit (Leica Biosystems, Buffalo Groves, IL, USA). The following antibodies were used: Aiolos monoclonal CELG977 antibody and Ikaros rabbit polyclonal antibody, respectively (Santa Cruz Biotechnology), c-Myc rabbit monoclonal (Abcam) and IRF4 mouse monoclonal antibody (Dako). Immunohistochemical immunoreactivity intensity was scored on a scale of 0–3 (0=negative, 1=weak, 2=intermediate and 3=strong). Percentages of cells with each intensity were recorded. H-score (0–300) was the sum of the products of each intensity value multiplied by the percentage of cells at that intensity to account for 100% of the tumor cells. *P*-values were determined by an unpaired *t*-test (two-tailed).

## Results

### Aiolos, Ikaros, c-Myc and IRF4 proteins are highly expressed in MM.

**Figure 1:**
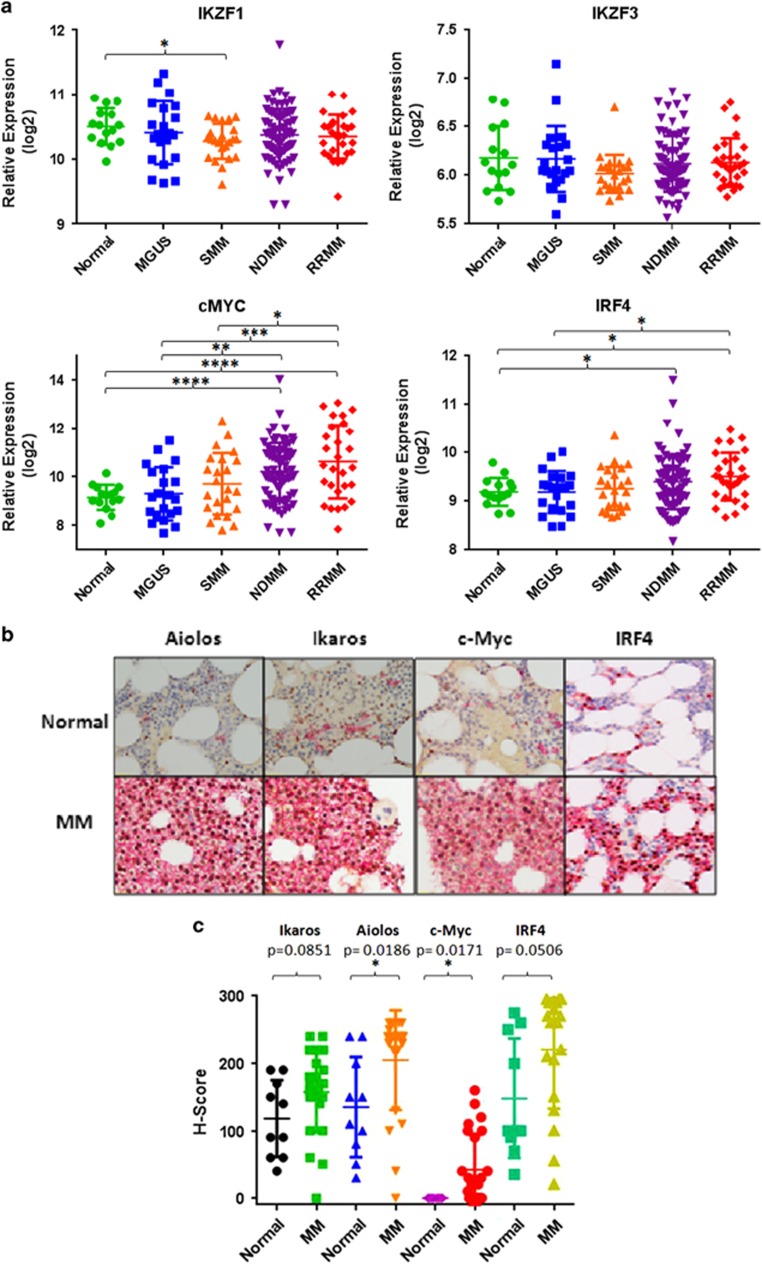
Ikaros, Aiolos, c-Myc and IRF4 are upregulated simultaneously in primary MM samples compared with normal bone marrow. (**a**) Microarray analysis of public data set GSE6477 showing the relative expression levels of *IKZF1*, *IKKZF3*, *c-Myc* and *IRF4* in normal (*n*=15), monoclonal gammopathy of undetermined significance (MGUS, *n*=21), SMM (*n*=23), newly diagnosed MM (NDMM, *n*=75) and relapsed/refractory MM (RRMM, *n*=28). (**b**) Dual-stained (CD138^+^, red; target protein, brown) immunohistochemical analysis of normal or MM bone marrow tissue for Ikaros, Aiolos, c-Myc or IRF4. (**c**) Compiled H-score analysis (see Materials and Methods) comparing normal (*n*=10) versus MM (*n*=24) bone marrow tissue microarrays for Ikaros, Aiolos, c-Myc or IRF4 by immunohistochemical assays represented in **b**. The Student's unpaired *t*-test was used to determine statistical significance, where *P<0.05, **P<0.01, ***P<0.001 and ****P<0.0001.

We analyzed the expression of *IKZF1*, *IKZF3*, *c-MYC* and *IRF4* genes from the public data set (GSE6477) of normal (*n*=15), monoclonal gammopathy of undetermined significance (*n*=21), smoldering MM (SMM, *n*=23), newly diagnosed MM (*n*=75) and relapsed/refractory MM (*n*=28) samples.^24^ *c-MYC* and *IRF4* expression markedly increased as the disease progressed from monoclonal gammopathy of undetermined significance to SMM, to newly diagnosed MM and relapsed/refractory MM (Figure 1a), consistent with dysregulation of their expression in the progression from normal to malignant state. In contrast, we did not observe significant change in the expression of either *IKZF1* or *IKZF3* genes during the progression from normal to monoclonal gammopathy of undetermined significance and SMM to newly diagnosed MM.

We previously described discordance between gene expression and protein measurement for CRBN in MM cell lines.^25^ Thus, we next analyzed the expression of Ikaros, Aiolos, c-Myc and IRF4 using analytically validated semi-quantitative immunohistochemical assays in primary bone marrow samples of normal (*n*=10) versus malignant (*n*=24) plasma cells by co-staining for CD138 and either Ikaros, Aiolos, c-Myc or IRF4, respectively (Figure 1b). All four transcription factors were overexpressed in MM compared with normal plasma cells, with c-Myc and Aiolos upregulation being statistically significant (Figure 1c). Although the increased gene expression and immunohistochemical measurements for c-Myc and IRF4 were consistent between the public data set (GSE6477)^24^ and the primary samples, we did not observe a similar correspondence for *IKZF1* or *IKZF3* gene expression with protein levels (Figure 1a). However, our immunohistochemical results may suggest an interesting possibility that increased levels of Ikaros and Aiolos could be linked to c-Myc and IRF4 overexpression in MM cells, extending their putative role in B-cell development as described previously.^3, 4^

### shRNA-mediated knockdown of IKZF1 or IKZF3 leads to c-Myc and IRF4 downregulation and is sufficient to inhibit proliferation and induce apoptosis in MM cells

**Figure 2:**
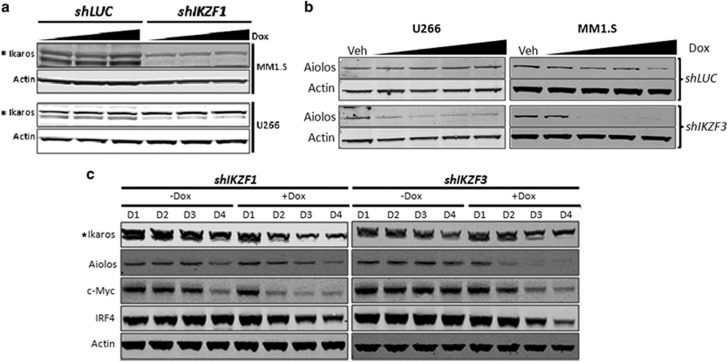
shRNA-mediated knockdown of *IKZF1* or *IKZF3* leads to the downregulation of c-Myc and IRF4. Decreased expression of Ikaros (**a**) or Aiolos (**b**) in stably transduced MM1.S and U266 MM cells after DOX induction (0.001–1 μg/ml) for 48 h of shRNAs targeting Ikaros (*shIKZF1*), Aiolos (*shIKZF3*) or luciferase as control (*shLUC*). (**c**) Western blot analysis of Ikaros, Aiolos, c-Myc and IRF4 in MM1.S *shIKZF1* or *shIKZF3* cells, which were cultured in the absence or the presence of DOX (Dox0.01 μg/ml), for 4 consecutive days (D1–D4). Asterik (*) by Ikaros indicates that only the bottom band was affected using this particular antibody (also see Materials and Methods).

Ikaros and Aiolos are degraded specifically in the presence of either lenalidomide or pomalidomide but not by other anti-myeloma agents such as dexamethasone, melphalan or bortezomib (Supplementary Figure S1a). To further investigate the dependence of MM cells on IKZF1 or IKZF3 expression for survival and elucidate the mechanism of action of lenalidomide and pomalidomide, we stably transduced lenalidomide- and pomalidomide-sensitive MM1.S and U266 cells for inducible expression of IKZF1 or IKZF3 shRNA (designated *shIKZF1* or *shIKZF3*, respectively). shRNA-mediated gene silencing allowed us to specifically analyze the effects of transcriptional silencing of *IKZF1* and *IKZF3*, uncoupled from the pleiotropic activities of lenalidomide or pomalidomide that may ensue from ubiquitination and proteasomal degradation of Ikaros and Aiolos. Compared with vehicle (Veh) or luciferase control shRNA (*shLUC*), *shIKZF1*- or *shIKZF3*-mediated knockdown reduced Ikaros or Aiolos protein levels by 60%–90% in the presence of DOX in both cell lines (Figures 2a and b). Furthermore, the knockdown of either Ikaros or Aiolos efficiently downregulated the expression of the other (Figure 2c), confirming the coordinated regulation of their respective gene expression in MM cells as previously described.^26, 27^

**Figure 3:**
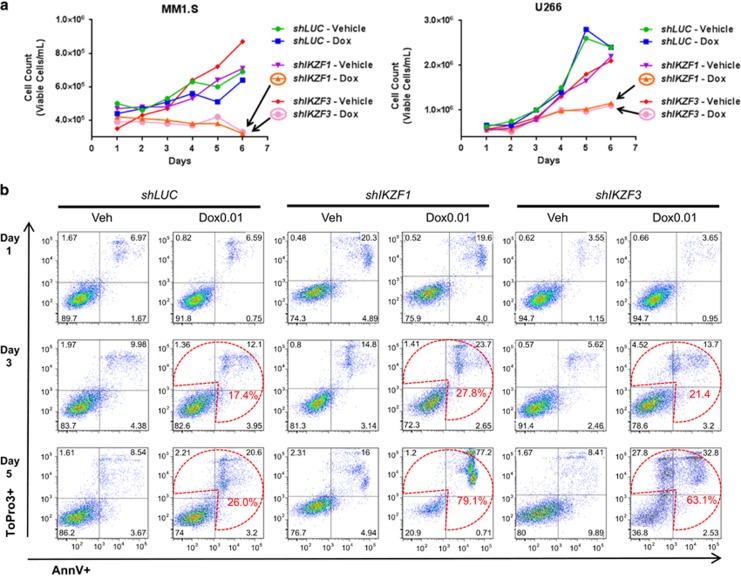
shRNA-mediated knockdown of *IKZF1* or *IKZF3* inhibits proliferation and induces apoptosis in MM cells. (**a**) Cell population growth chart of MM1.S (left panel) or U266 (right panel) following DOX-induced shRNA knockdown of either *IKZF1* or *IKZF3* as compared with Vehicle (Veh) or *shLUC* control. (**b**) Apoptosis measured by flow cytometry using Annexin-V (AnnV^+^; *x* axis) and ToPro3^+^ (*y* axis) staining following either Veh or DOX (Dox, 0.01 μg/ml) treatment of *shLUC* (left panel), *shIKZF1* (middle panel) or *shIKZF3* (right panel). MM1.S cells on day 1 (top row), 3 (middle row) and 5 (bottom row). Figures shown are representatives of *n*=3 experiments. Prominent increases in apoptotic cell numbers compared with *shLUC* or uninduced Veh control samples are indicated in red as % of total cell numbers representing the sum of the three quadrants enriched for apoptotic cells.

Degradation of Ikaros and Aiolos in the presence of lenalidomide was previously shown to correlate with the reduction of c-Myc and IRF4 levels in MM cell lines.^20^ We wanted to investigate whether the selective downregulation of *IKZF1* or *IKZF3* with shRNAs lead to reductions in c-Myc and IRF4 protein levels. Indeed, DOX-induced knockdown of *IKZF1* or *IKZF3* led to subsequent reductions in c-Myc and IRF4 levels. Over a 4-day time course, the reductions of c-Myc and IRF4 protein levels were maximal on day 4 (D4) (Figure 2c). These results suggest that reductions in either Ikaros or Aiolos were sufficient to downregulate c-Myc and IRF4 protein levels. In addition, cell growth (Figure 3a) and ^3^H-thymidine incorporation (Supplementary Figure S1B) were severely compromised in a time-dependent manner in response to either *IKZF1* or *IKZF3* knockdown. The reduction of Ikaros or Aiolos protein levels correlated with the induction of apoptosis as measured by AnnV^+^/ToPro3^+^ staining in MM1.S cells (Figure 3b). Although DOX treatment caused some apoptosis on its own in the *shLUC* control cells, DOX-induced Ikaros or Aiolos knockdown caused a substantial increase in apoptosis starting on day 3 (17.4 versus 27.8 or 21.4%) and further increasing on day 5 (26 versus 79.1 or 63.1%), respectively (Figure 3b). Similar results were obtained in U266 cells and using independent shRNAs (data not shown). Taken together, these results suggest that downregulation of either Ikaros or Aiolos was sufficient to inhibit proliferation and induce apoptosis in lenalidomide- and pomalidomide-sensitive cells.

### DOX-washout partially reverses the anti-proliferative effects of Ikaros and Aiolos knockdown

**Figure 4:**
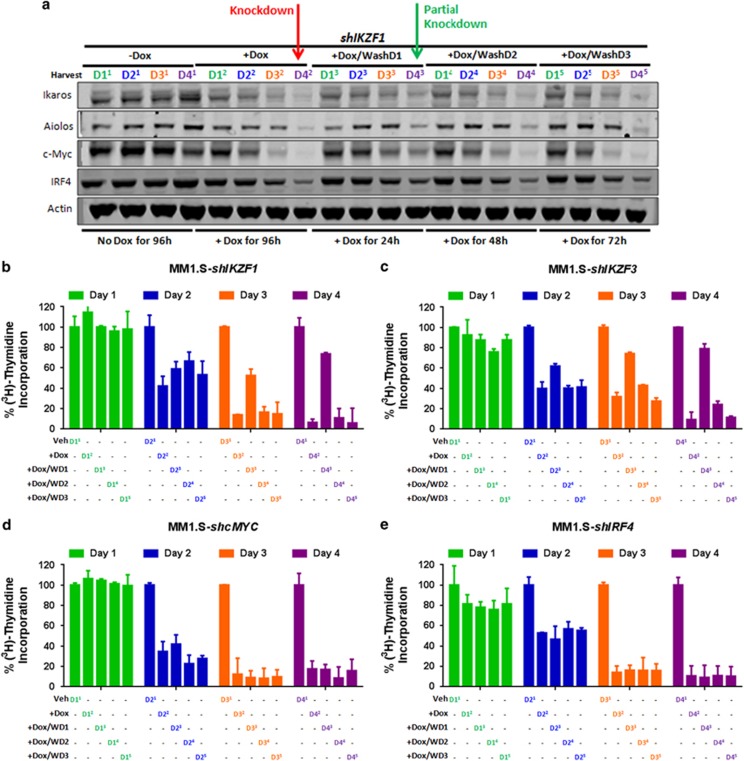
DOX washout of shRNA knockdown of *IKZF1* or *IKZF3* but not *c-MYC* or *IRF4* leads to a partial rescue of their respective anti-proliferative effects in MM cells. (**a**) Western blot analysis showing the relative expression of Ikaros, Aiolos, c-Myc and IRF4 in MM1.S *shIKZF1* cells cultured with or without DOX (Dox, 0.01 μg/ml) for 4 consecutive (D4) days in five distinct cultures marked with superscripts 1 through 5 (see Results section for details). The five parallel cultures consisted of culture 1 (−DOX), culture 2 (+DOX), culture 3 (DOX was washed out after day 1 (+DOX/WashD1)), culture 4 (DOX was washed out after day 2 (+DOX/WashD2)), or culture 5 (DOX was washed out after day 3 (+DOX/WashD3)), followed by continuous culture up to 4 days while the samples were collected each day (D1^1–5^ through D4^1−5^). Maximum protein reductions are visualized by D4 (red arrow), or partial knockdown (green arrow) following washout after D1. (**b**) Measurement of proliferation by ^3^H-thymidine incorporation in MM1.S *shIKZF1* cells in parallel samples to the experiment (see Results) described in part (**a**). Similar DOX-washout experiments as described in **a** followed by measurement of ^3^H-thymidine incorporation similar to part **b** were performed with *shIKZF3* (**c**), *shMYC* (**d**) or *shIRF4* (**e**) MM1.S cells.

We next sought to answer two important questions: (1) how long does the loss of Ikaros and Aiolos need to be sustained and (2) which step determines cellular commitment for the inhibition of proliferation and induction of apoptosis. To answer these questions, five parallel cultures were set up with or without DOX for 4 days (D4). Culture 1 (Figure 4; marked with superscript 1) was without DOX and culture 2 (superscript 2) was in the presence of DOX for 4 consecutive days. In cultures 3, 4 and 5 (superscripts 3, 4 and 5), DOX was washed out after D1, D2 or D3, respectively, followed by DOX-free growth until the end of D4. Aliquots from each culture were collected each day (D1–D4) to assess protein levels as well as proliferation in parallel. As shown in Figure 4a for *shIKZF1* knockdown, continuous exposure to DOX resulted in the gradual decrease in Ikaros levels, accompanied by concomitant reductions in Aiolos, c-Myc and IRF4, with the most pronounced effect being observed on D4 (red arrow). However, when DOX was removed after 24 h (D1), we observed a partial reduction in the amount of Ikaros protein by the end of D4, along with reductions in Aiolos, c-Myc and IRF4 (green arrow). Washout of DOX on D2 or D3 had similar effects to continuous treatment of DOX for 4 days. Proliferation was partially rescued after washout on D1, consistent with the partial reduction in protein levels on D4 but not after D2 or D3 washouts as shown in Figure 4b. Similar results were achieved with shRNA knockdown of *IKZF3* (Supplementary Figure S2A and Figure 4c). Conversely, DOX removal after D1 in *shMYC* MM1.S cells did not rescue c-Myc expression (Supplementary Figure S2B) nor its anti-proliferative effect (Figure 4d). Although DOX washout after IRF4 knockdown on D1 did recover IRF4 expression to some extent (Supplementary Figure S2C), there was no rescue in proliferation inhibition similar to that observed for c-MYC knockdown (Figure 4e). Collectively, these results suggest that the knockdown of *IKZF1* or *IKZF3* was required to be sustained in MM1S or U266 (data not shown) cells for more than 24 h, to achieve effective downregulation of c-Myc and IRF4 levels and to attain anti-proliferative activity in our experimental system. Second, they reveal that the ensuing downregulation of the c-Myc/IRF4 axis was a critical factor in the commitment to cell death downstream of Ikaros and Aiolos degradation triggered by lenalidomide or pomalidomide treatment.

### Kinetic analysis of shRNA knockdown of Ikaros or Aiolos reveals a temporal sequence of events in MM cells

**Figure 5:**
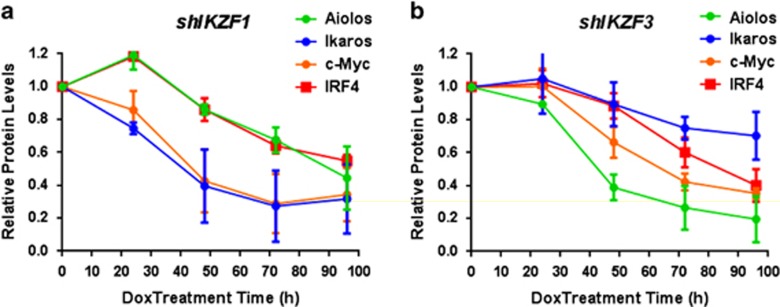
Temporal kinetics of shRNA-induced knockdown of Ikaros or Aiolos protein followed by downregulation of c-Myc and then IRF4 protein levels. Quantified protein expression levels from western blot analysis of Ikaros, Aiolos, c-Myc and IRF4 in MM1.S *shIKZF1* (**a**) or *shIKZF3* (**b**) cells, which were cultured in the absence or the presence of DOX (Dox, 0.01 μg/ml) for the indicated time points (24–96 h) were normalized to Actin levels as a loading control and plotted as a function of time follo*w*ing DOX-induced knockdown.

Having established a critical link between c-Myc and IRF4, and the anti-proliferative effects of Ikaros or Aiolos knockdown, we next analyzed the kinetics of shRNA knockdown of *IKZF1* or *IKZF3* with respect to the onset of c-Myc and IRF4 downregulation in MM1.S. As shown in Figure 5a, shRNA-mediated knockdown of Ikaros (blue line) became evident after 24 h of DOX treatment and reached a maximum decrease by 72–96 h. c-Myc levels (orange line) tracked nearly concurrently with the reduction of Ikaros levels. In contrast, there was a temporal delay in reaching the final extent of IRF4 (red line) and Aiolos (green line) reduction after sustained knockdown of Ikaros. Similarly, DOX-induced knockdown of Aiolos protein (Figure 5b) was followed by a considerable reduction in c-Myc and later reductions in IRF4 and Ikaros protein levels. Although there appeared to be differences between Ikaros and Aiolos knockdown in achieving the maximal reductions of c-Myc and IRF4 levels, the overall sequence remained consistent. Together, these results indicate that there is a temporal order in the reduction in c-Myc and IRF4 as a consequence of either *IKZF1* or *IKZF3* shRNA knockdown with c-Myc being more sensitive than IRF4 to the knockdown of either transcription factor. These results further suggest that IMiD drugs likely disrupt a functional link between c-Myc and IRF4 in MM cells via Ikaros and Aiolos degradation.

### Lenalidomide and pomalidomide differ in kinetics of Ikaros and Aiolos degradation

**Figure 6:**
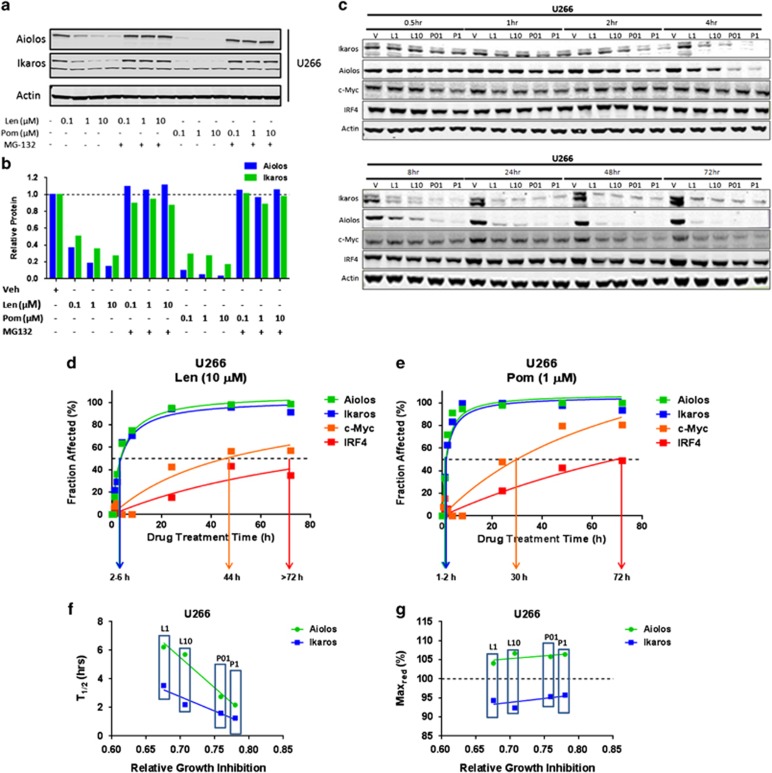
Differential kinetics of lenalidomide- or pomalidomide-induced degradation of Ikaros or Aiolos, followed by downregulation of c-Myc and IRF4. (**a**) Western blot analysis showing the reduction of Ikaros and Aiolos protein levels in U266 cells following treatment with increasing concentrations of either lenalidomide or pomalidomide for 6 h, which is abrogated in the presence of MG-132. (**b**) The data in **a** were quantified by densitometry, followed by normalization to the Actin loading control and graphed as fraction affected versus drug concentration. (**c**) U266 cells were treated with either vehicle (Veh), lenalidomide (L1 or L10 μM) or pomalidomide (P1 or P10 μM) for 0.5, 1, 2, 4, 8, 24, 48 and 72 h, and protein lysates were analyzed by western blotting for Ikaros, Aiolos, c-Myc and IRF4. (**d**) The data in **c** were quantified by densitometry, followed by normalization to the Actin-loading control and transformed into fraction affected. These data were then fit to a rectangular hyperbolic model (see Materials and Methods) to determine the time point at which 50% (*T*_1/2_) or the maximal amount (Max_red_) of the relative input protein amount is either degraded, as in the case of Ikaros or Aiolos, or downregulated, as for c-Myc or IRF4 in lenalidomide (10 μM) (**d**) or pomalidomide treated (1 μM) (**e**) in U266 cells. Calculated *T*_1/2_ (**f**) or Max_red_ (**g**) of Ikaros or Aiolos from either lenalidomide-treated (L1 or L10 μM) or pomalidomide-treated (P01 or P1 μM) cells plotted against the relative growth inhibition after 72 h for the indicated drug treatment.

In order to extend our observations from the kinetic experiments with shRNA knockdown of *IKZF1* or *IKZF3* to IMiD compound-mediated effects, we first investigated lenalidomide and pomalidomide concentration-dependent degradation of Ikaros and Aiolos. Treatment of U266 cells with either lenalidomide or pomalidomide in a range of concentrations (0.1–10 μM) for a fixed time course of 6 h showed a concentration-dependent degradation of both Ikaros and Aiolos (Figure 6a; top panel). As expected, these effects were abrogated in the presence of MG-132, a proteasome inhibitor (Figure 6a; top and bottom panels). As seen in Figure 6a (bottom panel), within 6 h there was a clear difference between lenalidomide and pomalidomide in the extent of Ikaros and Aiolos degradation. Pomalidomide appeared to be more efficient than lenalidomide in degrading either substrate across the range of concentrations tested. Based on these results, we chose concentrations for lenalidomide (1 or 10 μM) or pomalidomide (0.1 or 1 μM) for subsequent analysis, as they yielded the most comparable levels of substrate degradation.

Next, we analyzed the kinetics of relative protein abundance for Ikaros, Aiolos, c-Myc and IRF4, following treatment with lenalidomide or pomalidomide in U266 (Figure 6c) or MM1.S (Supplementary Figure S3) for 0.5, 1, 2, 4, 8, 24, 48 and 72 h. Degradation of Ikaros and Aiolos was evident within 2 h of drug treatment and the level of degradation increased over time and was sustained for the entire 72-h period. Measurable reduction of c-Myc and IRF4 was observed after the 24- to 48-h time points. We constructed kinetic models by using the relative abundance of proteins over time and drug concentrations, and then fitted them to a rectangular hyperbolic function (see Materials and Methods). An example of a kinetic model for lenalidomide is shown in Figures 6d and for pomalidomide in Figure 6e. These models revealed that the reduced levels of all four proteins over time had a strong goodness-of-fit to a hyperbolic curve. There was a clear difference in the kinetics of Ikaros and Aiolos relative abundance (Fraction affected) compared with that for c-Myc and IRF4. Ikaros and Aiolos were both rapidly degraded (*T*_1/2_=1–6 h; from pomalidomide to lenalidomide, respectively; Figures 6d and e) followed by a sustained plateau (Max_red_ approaches 100%) over 72 h for both lenalidomide or pomalidomide treatment. Conversely, c-Myc and IRF4 were downregulated with a substantial delay (*T*_1/2_=24–72 h; from pomalidomide to lenalidomide, respectively; Figures 6d and e) without reaching a plateau within this time frame. Again, there was a noticeable delay in IRF4 downregulation following c-Myc with both drugs, confirming the shRNA kinetic results above showing that c-Myc expression was more sensitive to the effects of Ikaros and Aiolos protein reduction. The delay in IRF4 downregulation could result from the initial delay in c-Myc downregulation, consistent with a proposed feedback loop between both transcription factors in MM.^9^ The rate (*T*_1/2_) of Ikaros or Aiolos degradation by either drug correlated well with relative growth inhibition by either drug, which appeared to be more sensitive to Aiolos than Ikaros degradation (Figure 6f). Unexpectedly, we found that the final extent (Max_red_) of substrate degradation did not correlate well with relative levels of growth inhibition caused by either drug at the concentrations tested (Figure 6g). However, pomalidomide had a greater effect than lenalidomide on the rate of either Ikaros or Aiolos degradation and this correlated with its greater growth inhibitory effect. Collectively, these data confirm the sequence of events observed with the shRNA knockdown experiments above. In addition, these results reveal a mechanistic difference between lenalidomide and pomalidomide in their relative efficiency around substrate degradation and downstream cytotoxic effects.

## Discussion

In the present report we explored the mechanistic role of Ikaros and Aiolos for the activities of lenalidomide and pomalidomide by using an inducible shRNA knockdown system. We performed kinetic analyses of degradation of both these ‘substrates of consequence' along with the downregulation of c-Myc and IRF4. Our results suggest that both Ikaros and Aiolos are crucial positive regulators of c-Myc and IRF4 expression in MM cells. In addition, we show that the upregulation of Ikaros and Aiolos appears to be pathologically associated with MM biology and the c-Myc/IRF4 axis. Finally, our shRNA data reveal that the anti-proliferative and pro-apoptotic activities of lenalidomide and pomalidomide differ in the relative efficiency of Ikaros and Aiolos degradation in IMiD compound-sensitive MM cells.

### Ikaros and Aiolos regulate c-Myc and IRF4 expression in MM

**Figure 7:**
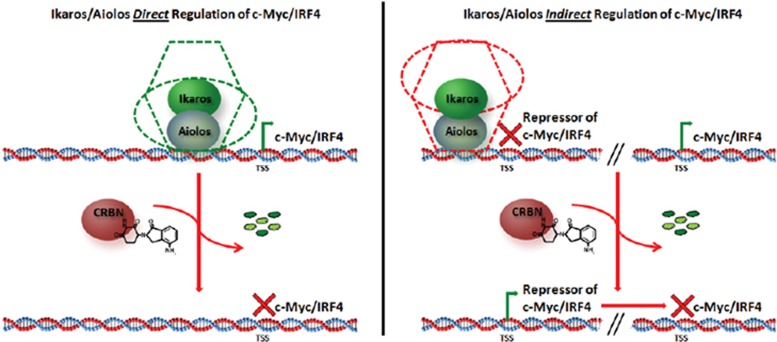
Graphical model explaining the potential mechanism of lenalidomide- or pomalidomide-negative regulation of the c-Myc/IRF4 axis. Two possible explanations for the negative regulation of the c-Myc/IRF4 axis either directly (left panel) or indirectly (right panel). For direct regulation, Ikaros/Aiolos are bound as *cis*-acting elements in a generic activating complex (green shape; that is, switch/sucrose nonfermentable (SWI/SNF)), followed by the lenalidomide- or pomalidomide-induced degradation of Ikaros/Aiolos and subsequent blockade of c-Myc/IRF4 transcription. For indirect regulation, Ikaros/Aiolos are bound as *trans*-acting elements in a generic repressive complex (red shape; that is, nucleosomal remodeling deacetylase (NuRD)) on an intermediate repressor of c-Myc/IRF4, followed by lenalidomide- or pomalidomide-induced degradation of Ikaros/Aiolos, expression of the intermediate repressor and subsequent blockade of c-Myc/IRF4 transcription.

Although our results showed the effect of either degradation or downregulation of Aiolos or Ikaros on the expression of c-Myc and IRF4, they did not address the mechanism by which Ikaros and Aiolos could regulate their expression. We consider two possible scenarios for the transcriptional regulation of c-Myc and IRF4. Figure 7 is a hypothetical model for either a direct (left side) or an indirect (right side) mode of regulation. A direct mode of action implies that Ikaros and Aiolos act as transcriptional activators of c-Myc and IRF4, as components of a transcriptional activation complex. Recent studies point to the physical occupation of the IRF4 promoter by Ikaros,^4^ suggesting that it may function as a transcriptional activator of IRF4 (Figure 7, left). However, chromatin immunoprecipitation–PCR analyses of Aiolos binding near the transcriptional start site of *c-Myc* or *IRF4* resulted in very few binding events (<4) compared with other target promoters (data not shown). Alternatively, it is possible that Ikaros and Aiolos negatively control the transcription of one or more intermediate (hitherto unknown) transcription factors that normally repress *c-Myc* and/or *IRF4* transcription. In this scenario, degradation of Ikaros and Aiolos would release the repression allowing expression of these repressors, which in turn will shut down the promoters of *c-Myc* and *IRF4* (Figure 7, right side). Indeed, as hematopoietic transcription factors, Ikaros and Aiolos are known to function within larger complexes, including the nucleosomal remodeling deacetylase complex, primarily acting as transcriptional repressors, and less frequently as components of the switch/sucrose nonfermentable complex that act as transcriptional activators.^28, 29^ A global loss of Ikaros and Aiolos, through targeted degradation, could therefore result in either the loss of positive activators or activation of negative intermediate transcriptional regulators, leading to the downregulation of the c-Myc/IRF4 axis in an indirect manner (Figure 7, right side). Additional investigation is necessary to address which mechanism is functionally operational in MM cells.

### Lenalidomide and pomalidomide are differentiated from each other by their relative effects on substrates in MM cells

Previous results have shown that lenalidomide and pomalidomide may have different thresholds for CRBN to drive their respective anti-proliferative activity.^2, 30^ Acquired resistance to lenalidomide resulted in lower expression levels of CRBN in MM cells, where pomalidomide was shown to retain anti-proliferative activity.^2^ In addition, crystal structure data shows subtle conformational changes around the IMiD immunomdulatory drug-binding pocket of CRBN between lenalidomide and pomalidomide.^31^ In this report, we demonstrate yet another level of differentiation based on the rate at which substrates are degraded in the presence of either lenalidomide or pomalidomide. Our kinetic modeling revealed that the rate but not the extent of lenalidomide- or pomalidomide-induced degradation of Ikaros and Aiolos correlates to the anti-proliferative activity of the drugs (Figure 6). Essentially, the shorter the time required to achieve 50% relative abundance of either Ikaros or Aiolos for pomalidomide and lenalidomide (<1 versus >3 h), the faster the rates of c-Myc (24 to >60 h) and IRF4 (36 to >72 h) downregulation and the greater the inhibition of proliferation that are observed (Figure 6f). This occurred by comparing increasing doses of either lenalidomide or pomalidomide, or by comparing lenalidomide versus pomalidomide, with the anti-proliferative and pro-apoptotic effects being greater with pomalidomide. This is unexpected, as anti-proliferative effects and induction of apoptosis occur at much later time points where the cumulative effects on Ikaros and Aiolos levels are similar (that is, Max_red_; Figure 6e and Supplementary Figure S3G). Even though the apparent differences between the rate of degradation of Ikaros and Aiolos (that is, *T*_1/2_; Figure 6f and Supplementary Figure S3F) seem modest between lenalidomide and pomalidomide, they appear significant enough to account for a more rapid depletion of c-Myc/IRF4 with increasing doses or with pomalidomide compared with lenalidomide, thus resulting in more pronounced cellular toxicity. A threshold of 50% reduction in c-Myc levels is achieved at >80% Aiolos/Ikaros downregulation after ~24 h with pomalidomide and 48 h with lenalidomide, respectively (Figures 6d and e). Nevertheless, despite the 50% c-Myc reduction, lenalidomide never results in similar growth inhibition and apoptosis as observed with pomalidomide, which reaches a similar extent considerably earlier (Figures 6d and e). This is also reflected by the fact that 50% downregulation of IRF4 levels, which may be required for a widespread apoptotic response, is barely reached, if at all, with lenalidomide at even much later time points. Importantly, we hypothesize that a delayed onset of response may provide the tumor cells with time to mount compensatory effects that attenuate the effect on growth and survival despite a similar final reduction in Ikaros and Aiolos levels. These data also provide an explanation of the difference in efficacy between the two compounds, despite having a similar binding affinity for CRBN.^2^ One possibility is that the turnover of substrates (the catalytic efficiency of the E3 ligase) is more efficient for pomalidomide-bound E3 ligase compared with that of lenalidomide-bound E3 ligase.

### Ikaros and Aiolos may be involved in the disease-promoting activity of c-Myc and IRF4 in MM

Both c-Myc and IRF4 are known oncogenes in MM, owing to their ability to support growth and survival.^7, 9^ Although the molecular mechanisms of c-Myc and IRF4 expression is complex, our findings here suggest that Ikaros and Aiolos are involved in the regulation of their transcriptional activity. Indeed, degradation of either Ikaros or Aiolos appear to significantly disrupt the reported feedback loop^9^ between c-Myc and IRF4. This disruption in turn likely affects downstream cellular effects on proliferation and apoptosis as indicated by the individual knockdown of either c-Myc or IRF4 (Figures 6d and e, and data not shown). Thus, Ikaros and Aiolos may be part of a disease-promoting/sustaining molecular transcriptional network in MM. Both Ikaros and Aiolos are known regulators of lymphoid and myeloid lineage development.^15, 16, 28, 29, 32^ During normal B-cell activation and maturation, the c-Myc/IRF4 regulatory axis is only transiently active during germinal center class switch recombination and in the expansion phase after normal B-cell activation.^10, 13, 14^ Subsequently, c-Myc becomes downregulated to allow for differentiation to the plasma cell stage. In agreement with this, in normal mouse B-cell development Ikaros and Aiolos have been shown to act as negative regulators of c-Myc expression in order to promote maturation and inhibit large pre-B-cell expansion.^16, 33^ By contrast, here we show that shRNA knockdown of Ikaros or Aiolos results in the downregulation of c-Myc and IRF4 expression (Figures 3 and 4), suggesting that there is a switch from a negative to a positive regulatory role in MM disease (Figure 7). The likely pathological rewiring of the Aiolos/Ikaros/c-Myc/IRF4 expression is further substantiated by the increased levels of c-Myc and IRF4 detected in CD138^+^ MM compared with normal plasma cells from bone marrow samples in the presence of elevated levels of Aiolos and Ikaros (Figures 1a and b). This type of functional switch is not without precedence. The conversion from an inhibiting to a tumor growth-promoting role has been described for the tumor suppressors Smad2, 3 and 4, which also function as transcriptional repressors.^34^

### Therapeutic implications

Our work has therapeutic implications derived from the observation that both c-Myc and IRF4 expression levels steadily increase over the course of MM disease progression from monoclonal gammopathy of undetermined significance to SMM, then newly diagnosed MM, and finally relapsed/refractory MM (Figure 1a), suggesting that the upregulation of this axis contributes to the pathogenesis of the disease. It is tempting to speculate whether it may be possible to halt or stall the progression of the disease, by targeting the c-Myc/IRF4 axis through early therapeutic intervention at the stage of asymptomatic SMM. In our studies, we show that lenalidomide or pomalidomide could effectively target the c-Myc/IRF4 axis, and that once that axis is compromised, MM cells can no longer be rescued from growth inhibition (Figure 4). In addition, our results and those of others^7, 8, 9, 17^ suggest that the c-Myc/IRF4 axis together with Ikaros and Aiolos may represent the so-called ‘Achilles heel' in MM, and through rational combinations of IMiD immunomodulatory drugs with targeted inhibitors of c-Myc it may be possible to explore beneficial therapeutic opportunities for patients.
