# Supplementary material for: Rate of CRL4CRBN substrate Ikaros and Aiolos degradation underlies differential activity of lenalidomide and pomalidomide in multiple myeloma cells by regulation of c-Myc and IRF4
Source: Blood Cancer J. 2015 Oct 2;5(10):e354–. doi: 10.1038/bcj.2015.66 (PMC4635186; doi:10.1038/bcj.2015.66)
Supplement: Supplementary Information [file bcj201566x1.pdf]

Supplementary Table 1

shRNA	Hairpin Sequence
shIKZF1-1	ACCGG***CGGTCTGAGGAATAACCAAAC*** GTTAATATTCATAGCGTTTGGTTGTTCCTCAGACCGTTTT
shIKZF1-2	ACCGG***GCTGGCTAATTAGTTATGTGA*** GTTAATATTCATAGCTCACATAGCTAATTAGCCAGCTTTT
shIKZF3-1	ACCGG***CGTGATGGACTAAGCCATTAA*** GTTAATATTCATAGCTTGATGGCTTGGTCCATCACGTTTT
shIKZF3-2	ACCGG***CCATCCGTGTTATGATATGGA*** GTTAATATTCATAGCTCCATGTCATAGCACGGATGGTTTT
shcMYC-1	ACCGG***CCTGAGACAGATTAGCAATAA*** GTTAATATTCATAGCTTGTTGCTGATCTGTCTCAGGTTTT
shcMYC-2	ACCGG***CAGTTGAAACACAAACTTGAA*** GTTAATATTCATAGCTTCAAGTTTGTGTTTCAACTGTTTT
shIRF4-1	ACCGG***GCGCTTTGAATAAGAGTAATG*** GTTAATATTCATAGCCATTGCTCTTGTTCAAAGCGCTTTT
shIRF4-2	ACCGG***CGGATGCTCTATTTCAATTGT***GTTAATATTCATAGCGCAATTGAAATGGAGCATCCGTTTT

Supplementary Table 1: List of shRNA hairpin sequences used within this study. At least 2 shRNA constructs for each target were utilized throughout the entirety of these studies, yet for simplicity, the data from the first listed shRNA sequence for each target are represented in all figures. Targeting sequence is italicized in bold. Induction of shRNA expression was done with either a titrating dose of doxycycline (Sigma) or a single, toxicity-limiting dose (10 ng/mL). For doxycycline washout experiments, cells were exposed to a single dose of doxycycline for the indicated times, followed by centrifugation and supernatant removal, washed once with culture media, and then replenished with fresh culture media without doxycycline.

Supplementary Figure 1

(A)

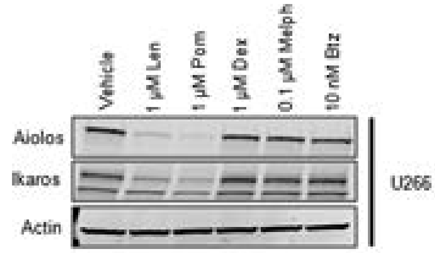

(B)

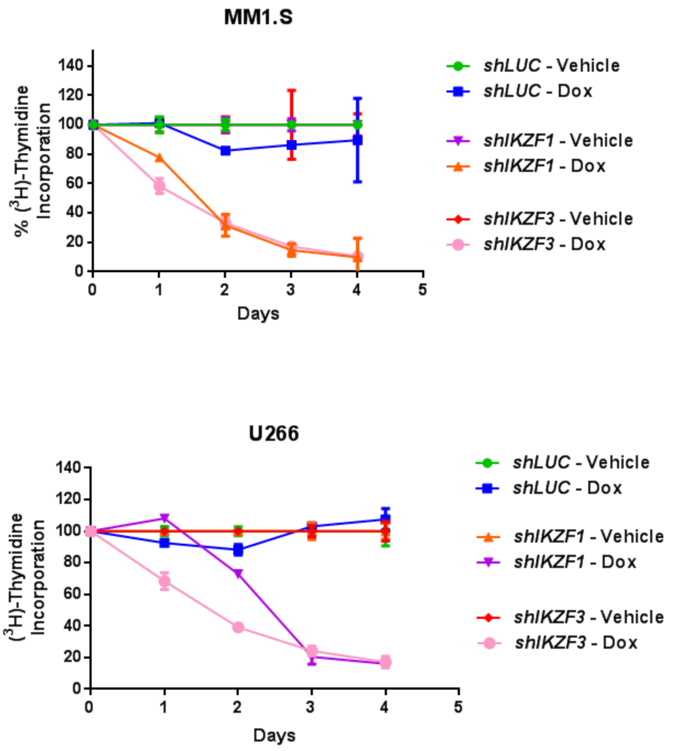

Supplementary Figure 1: Knockdown of Ikaros or Aiolos slows proliferation in MM cells. (A) Immunoblot analysis after 24 hr treatment with either lenalidomide (1 µM), pomalidomide (1 µM), dexamethasone (1 µM), melphalan (0.1 µM) or bortezomib (10 nM), showing that Ikaros and Aiolos degradation in U266 MM cells is unique to lenalidomide and pomalidomide. **(B)** ^{3}H-thymidine incorporation of MM1.S (top panel) or U266 (bottom panel) following either vehicle (Veh) or doxycycline (Dox0.01 µg/mL) treatment of shLUC, shIkaros1 and shAiolos1 over 4 days. Figures shown are representatives of n = 3 experiments.

Supplementary Figure 2

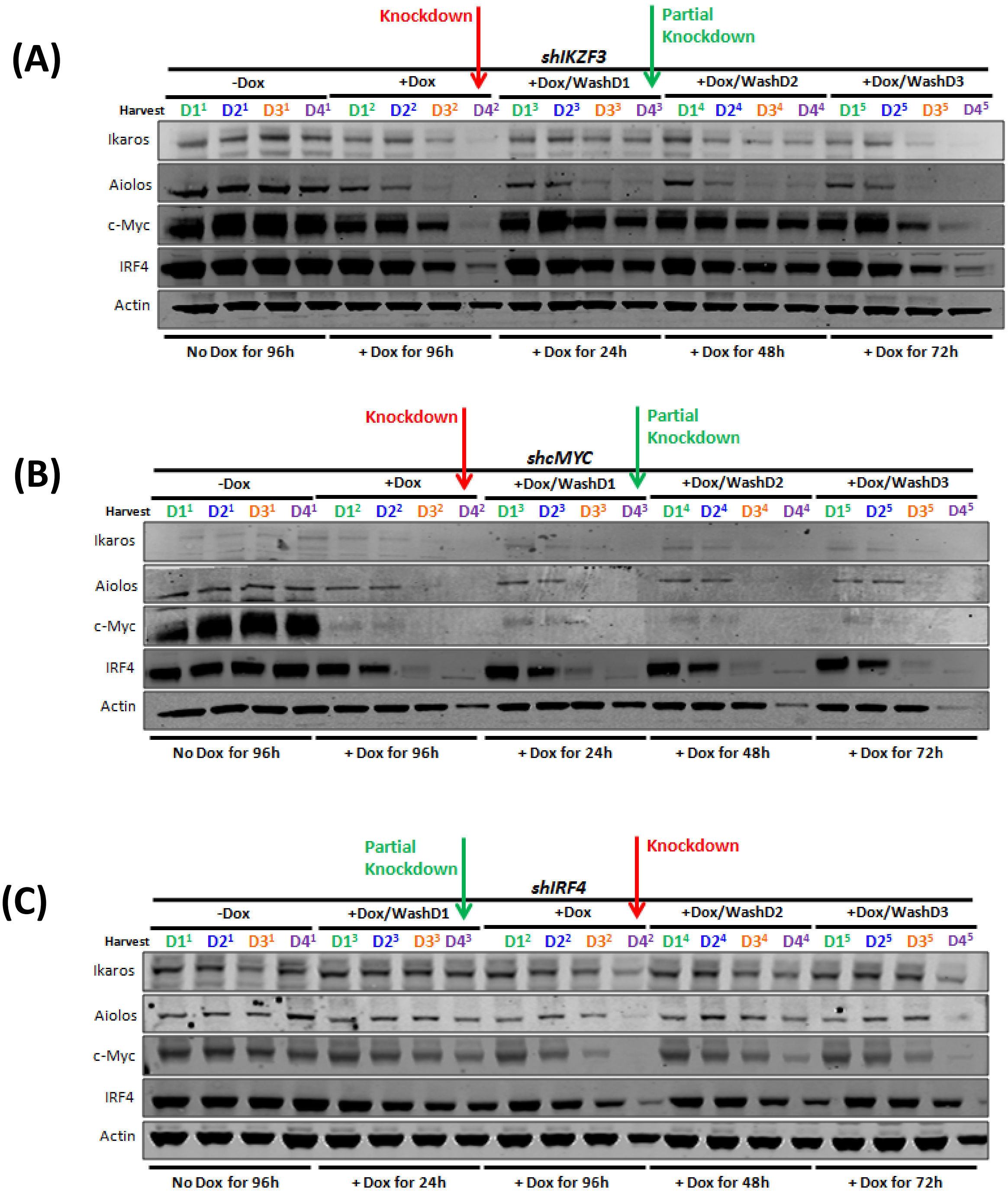

Supplementary Figure 2: Doxycycline-washout of shRNA knockdown of IKZF1 or IKZF3, but not cMYC or IRF4 leads to a partial rescue of their respective anti-proliferative effects in MM cells. Western blot analysis showing the relative expression of Ikaros, Aiolos, c-Myc and IRF4 in MM1.S *shIKZF3* **(A)**, *shcMYC* **(B)**, *or shIRF4* **(C)** cells cultured with or without DOX (Dox0.01 µg/mL) for 4 consecutive (D4) days in five distinct cultures marked with superscripts 1 through 5 (see results section for details). The five parallel cultures consisted of culture (-DOX), culture 2 (+DOX), culture 3 (DOX was washed out after day 1 (+DOX/WashD1)), culture 4 (DOX was washed out after day 2 (+DOX/WashD2)), or culture 5 (DOX was washed out after day 3 (+DOX/WashD3)), followed by continuous culture up to four days while the samples were harvested each day ($D1^{1-5}$ through $D4^{1-5}$). Maximum protein reductions are visualized by day 4 (red arrow), or partial knockdown (green arrow) following washout after day 1. Notice the two groups are switched for *shIRF4* **(B).**

Supplementary Figure 3

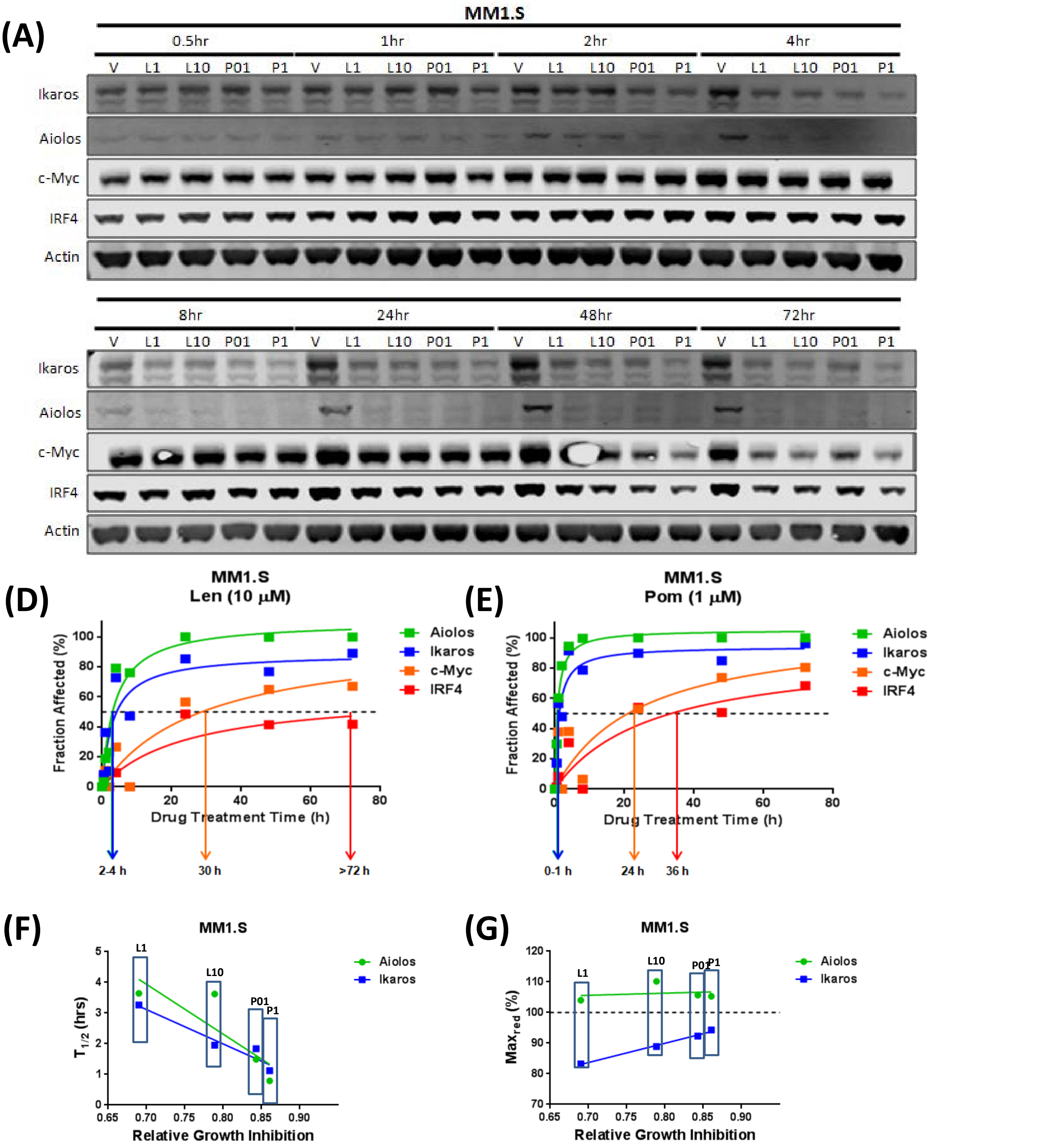

Supplementary Figure 3: Differential kinetics of lenalidomide- or pomalidomide-induced degradation of Ikaros or Aiolos, followed by downregulation of c-Myc and IRF4. (A) MM1.S cells were treated with either vehicle (Veh), lenalidomide (L1 or L10 μM) or pomalidomide (P1 or P10 μM) for 0.5, 1, 2, 4, 8, 24, 48, and 72 hrs and protein lysates were analyzed by Western blot for Ikaros, Aiolos, c-Myc and IRF4. **(B)** The data in part **(C)** was quantified by densitometry, followed by normalization to the Actin loading control and transformed into fraction affected. These data were then fit to a rectangular hyperbolic model (see Methods) to determine the time point at which 50% ($T_{1/2}$), or the maximal amount (Max_{red}) of the relative input protein amount is either degraded, as in the case of Ikaros or Aiolos, or downregulated, as for c-Myc or IRF4 in lenalidomide- (10 μM) **(D)**, or pomalidomide-treated (1 μM) **(E)** in MM1 cells. Calculated $T_{1/2}$ **(F)** or Max_{red} **(G)** of Ikaros or Aiolos from either lenalidomide-treated (L1 or L10 μM), or pomalidomide-treated (P01 or P1 μM) cells plotted against the relative growth inhibition after 72 hours for the indicated drug treatment.
